# Low dose combined oral contraceptives induced thrombotic anterior wall myocardial infarction: a case report

**DOI:** 10.1186/s12872-020-01462-9

**Published:** 2020-04-19

**Authors:** Alaa Rahhal, Fadi Khir, Mohammad Adam, Amer Aljundi, Mohammed Khalil Mohsen, Jassim Al-Suwaidi

**Affiliations:** 1 Pharmacy Department, Heart Hospital, Hamad Medical Corporation, P.O. Box 3050, Doha, Qatar; 2 Internal Medicine Department, Hamad General Hospital, Hamad Medical Corporation, Doha, Qatar; 3 Cardiology and Cardiovascular Surgery Department, Heart Hospital, Hamad Medical Corporation, Doha, Qatar

**Keywords:** Case report, Oral contraceptives, Myocardial infarction

## Abstract

**Background:**

Combined oral contraceptive pills are associated with an established risk for venous thrombosis; however, their risk for arterial thrombosis remains uncertain, especially with the development of low dose new generations of combined oral contraceptive. Arterial thrombosis is less likely to occur with the use of oral contraceptive pills in the absence of cardiovascular risk factors.

**Case presentation:**

We report a 35-year old female with no cardiovascular risk factors who presented with thrombotic anterior wall myocardial infarction 6 months after using a third generation low dose combined oral contraceptive pills (Marvelon; ethinylestradiol 30 mcg and desogestrel 150 mcg).

**Conclusion:**

Third generation low dose combined oral contraceptives may lead to myocardial infarction in young women, even in the absence of other cardiovascular risk factors.

## Background

Since the development of the oral contraceptive (OC) pills, their association with an increased risk of venous thromboembolism has been well established. This risk has been decreased, although not yet eliminated, by the introduction of newer generations of oral contraceptives with reduced doses of estrogen. The risk of arterial thrombosis has been a universally feared, but not well established, adverse event of the oral contraceptives. This risk is thought to be cumulative with the association of other risk factors of arterial thrombosis [1].

In this case report, we present a young lady who was admitted to our hospital with the diagnosis of an ST-elevation myocardial infarction (STEMI). Apart from the use of a third generation of OC pills, she has no associated cardiovascular risk factors.

## Case presentation

A 35-year-old female with no past medical history was admitted to the Heart Hospital in Qatar, a cardiology-specialized facility, with typical chest pain that began 2 h before admission. She described the pain as a pressure-like sensation radiating to her left arm and back and was associated with sweating. She was not a smoker or alcohol consumer and reported no illicit drug use; however, laboratory confirmation was not pursued as the suspicion of drug abuse was low. She had no history of miscarriages and no family history of coronary artery disease. She was married and using a third generation low dose combined oral contraceptive, Marvelon (ethinylestradiol 30 mcg and desogestrel 150 mcg), for 6 months.

Her vital signs and body mass index were within normal range, as follows: heart rate: 71/min, blood pressure: 126/73 mmHg, respiratory rate: 17/min, oxygen saturation on room air: 100%, and body mass index: 23 Kg/m2.

**Fig. 1:**
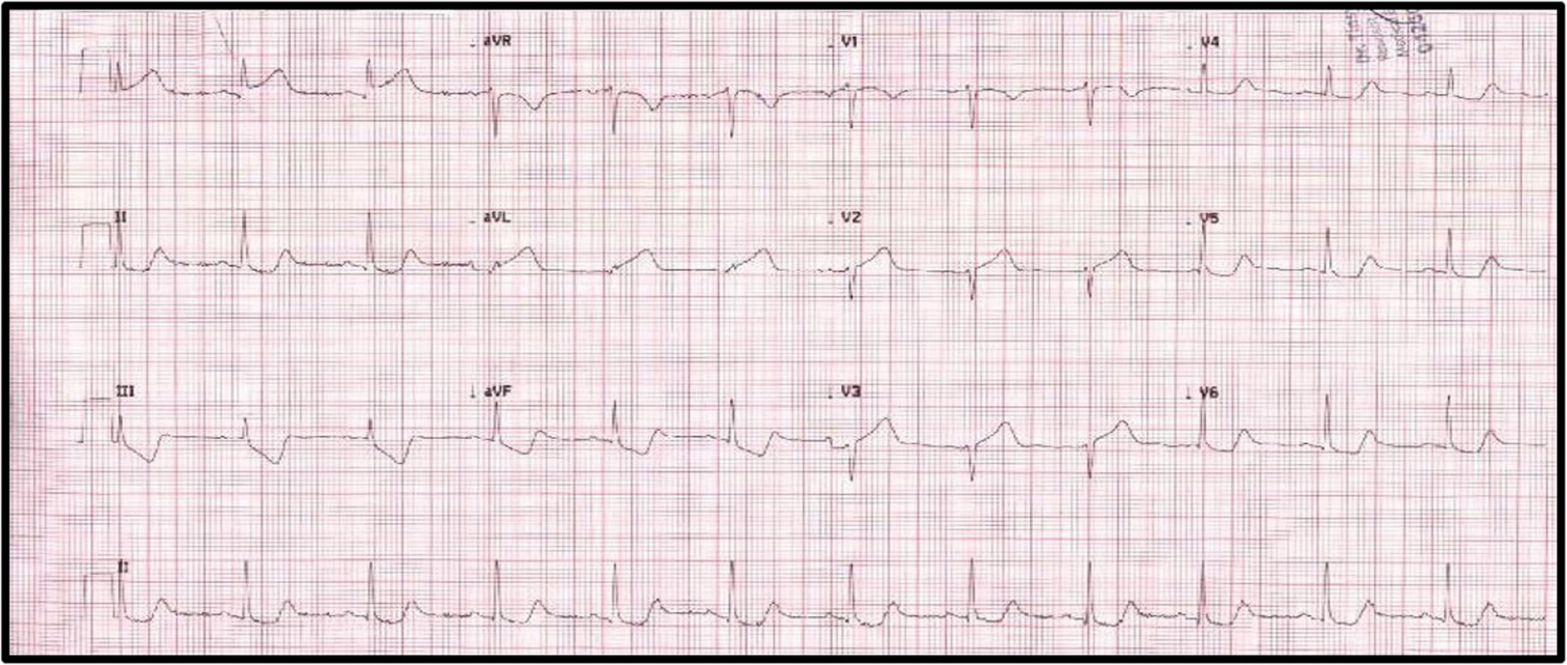
Electrocardiographic features of acute anterior wall myocardial infarction at presentation

**Fig. 2:**
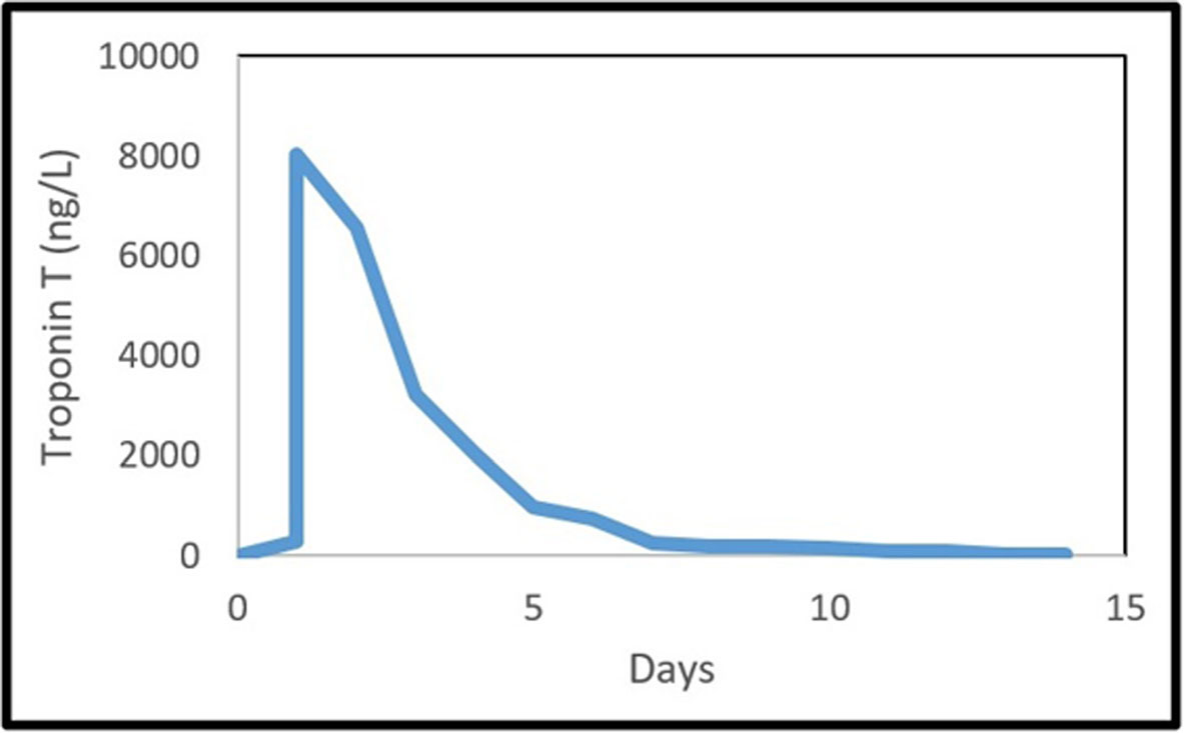
High sensitive Troponin T trend during hospitalization

Upon admission, the 12-lead electrocardiogram (ECG) showed ST-segment elevation mainly in leads I and aVL in addition to minimal elevation in V2 and V3 and associated poor R wave progression as shown in Fig. 1. The patient’s cardiac enzymes (high sensitive Troponin-T) was elevated upon admission, and it continued to rise in the first day to 8026 ng/L as demonstrated in Fig. 2.

**Fig. 3:**
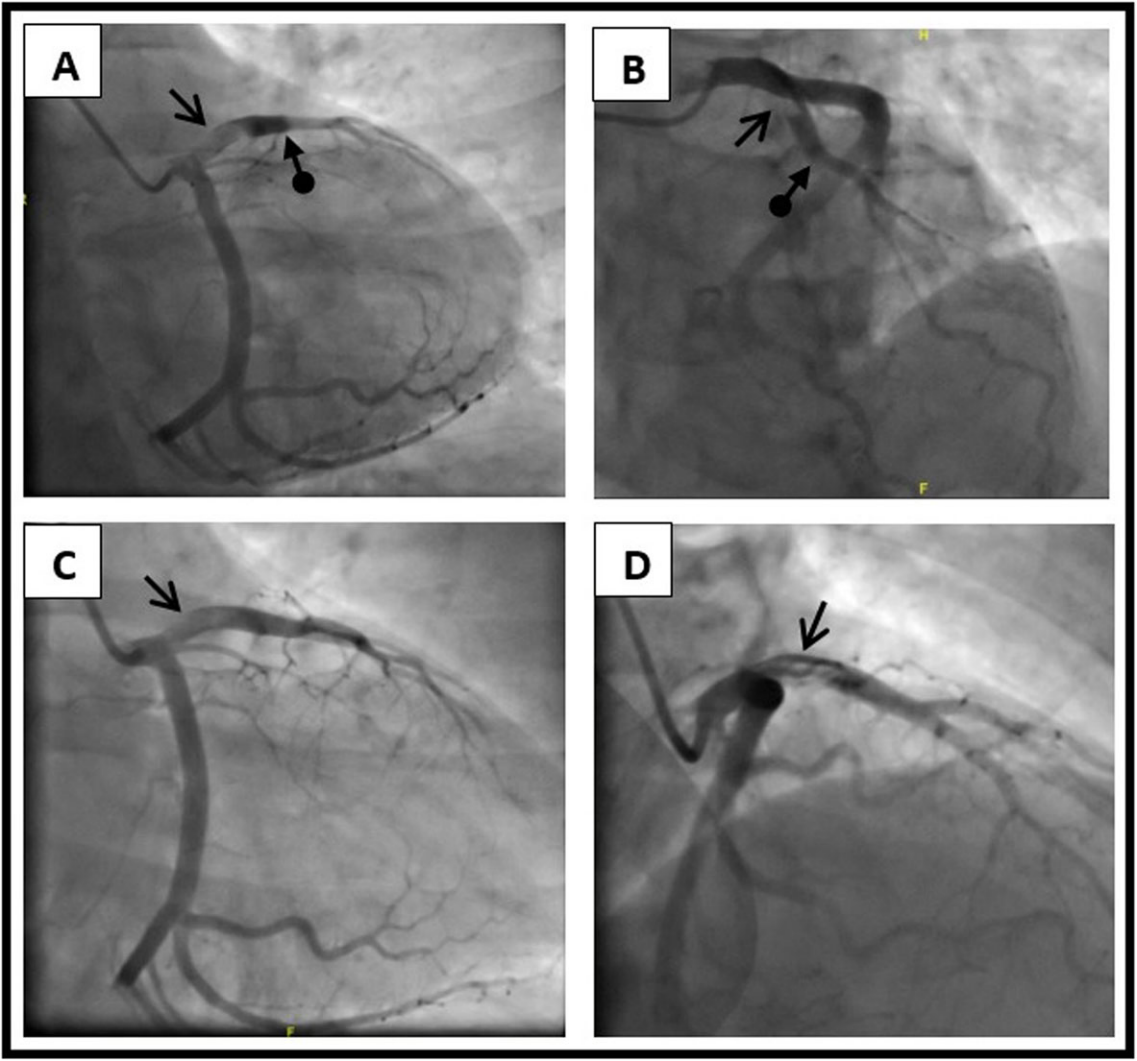
**a** and **b**: Angiographic imaging of the proximal LAD thrombus and mid LAD occlusion before thrombus aspiration in the right caudal oblique projection and left cranial oblique projection; respectively. **c** and **d**: Angiographic imaging of LAD after thrombus aspiration in the right caudal oblique projection and right cranial oblique projection; respectively

Primary percutaneous coronary intervention (PCI) was performed and showed a big thrombus in the proximal left anterior descending (LAD) artery and an occlusion in the mid LAD. Thrombus aspiration was done, and post aspiration coronary angiography showed residual thrombus, however, TIMI II- III flow was established, as demonstrated in Fig. 3.

She was admitted to the Cardiac Intensive Care Unit (CICU) with 48 h of eptifibatide infusion along with dual antiplatelet agents (aspirin and clopidogrel) as a case of thrombotic anterior wall myocardial infarction. Her echocardiogram showed hypokinesia of the antero-septal area with akinesia of the apical region. Ejection fraction (EF) was estimated to be 48%. Thrombophilia workup was negative, including lupus anticoagulant, protein S and protein C. Protein C activity was 103.5% (70–140) and protein S activity was 66.5% (56–126). Autoimmune disease screening was negative, including rheumatoid factor and antinuclear antibody (ANA).

**Fig. 4:**
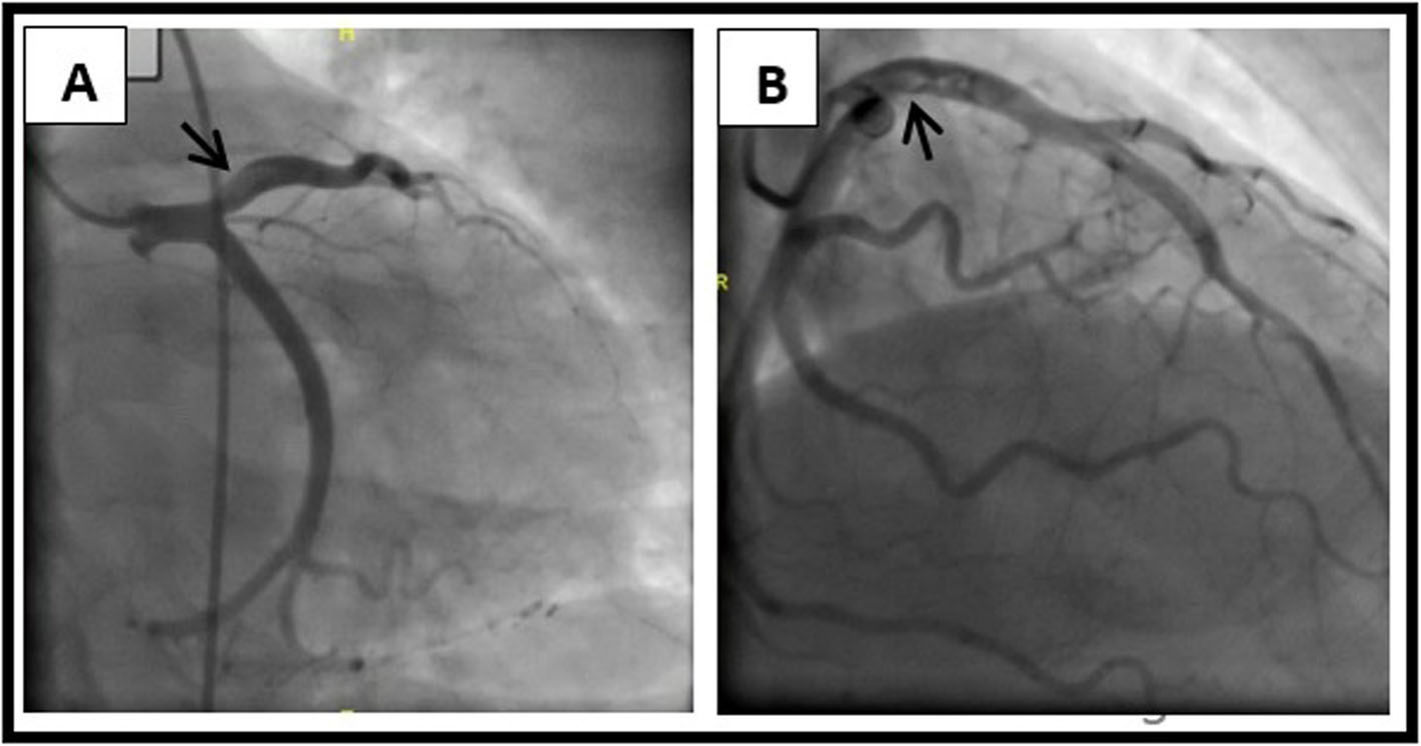
**a** and **b**: Angiographic imaging of the residual thrombus at proximal LAD in the right caudal projection and right cranial oblique projection; respectively

On the fifth day of admission, she had re-look coronary angiography (CAG) which showed residual thrombus again, with no change in size, as shown in Fig. 4. However, thrombus aspiration was not performed because of risk of distal embolization. Therefore, she was kept on therapeutic enoxaparin at a dose of 1 mg/kg subcutaneous twice daily until her Troponin-T normalized and discharged home on day 17th of admission. The patient was seen in the follow-up clinic 2 months post discharge and was found to be asymptomatic.

## Discussion and conclusion

Thrombosis, venous as well as arterial, is the most frequently occurring serious side effect of combined oral contraceptives [1]. To reduce the harmful thrombotic risks of oral contraceptives, over the past decades, the hormonal components of OC pills have been modified. The estrogen dose has been reduced from 150 mcg to less than or equal to 30 mcg, and new generations of progestin hormone were developed [2]. Combined oral contraceptives in the 1960s contained a first-generation progestin (norethisterone, lynestrenol), then in the 1970s, the second generation (levonorgestrel, norgestrel) was used, and in the 1980s and 1990s, the third generation (desogestrel, gestodene) started to be used in order to reduce the androgenic side effects [3–5].

The association between oral contraceptives and venous thrombosis is well established with an estimated risk of three to six folds increase compared to non-OC users [1]. Nevertheless, the association of oral contraceptives with arterial thrombosis, including myocardial infarction (MI), is controversial and not yet well-established. A recent Cochrane meta-analysis of 24 studies showed that oral contraceptives increased the risk of arterial thrombosis, including MI or ischemic stroke by 1.6 folds; MI (Relative Risk [RR] 1.6, 95% CI 1.2 to 2.1) and ischemic stroke (RR 1.7, 95% CI 1.5 to 1.9). Interestingly, the risk of arterial thrombosis did not vary among the different progestin generations; however, the relative risk increased with increasing the estrogen dose. For preparations containing 20 mcg of estrogen the relative risk is 1.6 (95% CI 1.4 to 1.8), for preparations containing 30 to 49 mcg of estrogen the relative risk is 2.0 (95% CI 1.4 to 3.0), and for preparations containing > 50 mcg of estrogen, the relative risk increases to 2.4 (CI 1.8 to 3.3) [6]. However, the risk of arterial thrombosis in relation to oral contraceptives (RATIO) study demonstrated that risk of MI associated with oral contraceptives is 2 (95% CI 1.5–2.8) and the risk was reduced with the third generation of progestin with an adjusted odds ratio of 1.3 (95% CI 0.7–2.5) [7].

The risk of oral contraceptive induced arterial thrombosis is more pronounced with other risk factors for arterial thrombosis; including smoking, hypertension, diabetes, and hypercholesterolemia [1]. It was shown that the risk of MI increased to 13.6-fold (95% CI 7.9–23.4) for OC users with smoking history, 6.1-fold (95% CI 3.1–12.1) for OC users with hypertension, 17.4-fold (95% CI 3.1–98.1) for OC users with diabetes, and 24.7-fold (95% CI 5.6–108) for OC users with dyslipidemia [7]. Age is a strong risk factor for thrombosis; however, it was shown that the risk of MI induced by oral contraceptives is more common in smoking women over 35 years of age [8]. Although, the mechanism of oral contraceptives induced MI is not well understood, the cause of MI among OC users is thrombotic rather than atherosclerotic and could be attributed to the pro-thrombotic effects of oral contraceptives [8]. Oral contraceptives are associated with increased coagulation factors, including factor VII, VIII, and X along with increased activity of the fibrinolytic inhibitors; Plasminogen Activator Inhibitor (PAI)-1 and PAI-2 and decreased levels of the natural anticoagulants; antithrombin and protein S [9, 10].

We reported a 35-year old lady who presented with anterior wall MI and was using a third generation low dose combined oral contraceptive for 6 months. She did not have any risk factors for coronary artery disease, including hypertension, diabetes, dyslipidemia, smoking, obesity, or family history for cardiovascular diseases. No hypercoagulable disorders or auto-immune diseases were identified.

To our knowledge, this is the second case report of low dose third generation oral contraceptives induced arterial thrombosis in the absence of risk factors for MI. Aslan AN et al. reported a case of a 20-year old female, non-smoker and with no risk factors for cardiovascular disease, who developed an inferior wall MI 1 month after the use of third generation low dose oral contraceptives [11]. Compared to that case, our patient was slightly older and had been using oral contraception for a longer period. Moreover, our patient developed anterior wall MI, while in the previously reported case, the patient presented with inferior wall MI. In our case, Thrombophilia and autoimmune workup was negative; however, this was not addressed in the above-mentioned case.

In conclusion, arterial thrombotic events such as myocardial infarction can occur among patients taking third generation combined oral contraceptive pills, even in the absence of risk factors for arterial thrombosis. With the widespread use of OC pills, we believe that physicians should keep this risk in mind when prescribing oral contraceptives.

## Data Availability

The datasets used and/or analyzed during the current study are available from the corresponding author on reasonable request.

## Abbreviations

OCP: Oral contraceptives
STEMI: ST-elevation myocardial infarction
ECG: Electrocardiogram
PCI: Percutaneous coronary intervention
LAD: Left anterior descending
CICU: Cardiac intensive care unit
EF: Ejection fraction
ANA: Antinuclear antibody
CAG: Coronary angiography
RR: Relative risk
